# Single-Shot 10K Proteome Approach: Over 10,000 Protein Identifications by Data-Independent Acquisition-Based Single-Shot Proteomics with Ion Mobility Spectrometry

**DOI:** 10.1021/acs.jproteome.2c00023

**Published:** 2022-05-06

**Authors:** Yusuke Kawashima, Hirotaka Nagai, Ryo Konno, Masaki Ishikawa, Daisuke Nakajima, Hironori Sato, Ren Nakamura, Tomoyuki Furuyashiki, Osamu Ohara

**Affiliations:** † Department of Applied Genomics, Kazusa DNA Research Institute, Kisarazu, Chiba 292-0818, Japan; ‡ Division of Pharmacology, Graduate School of Medicine, Kobe University, Chuo-ku, Kobe 650-0017, Japan

**Keywords:** single-shot proteomics, DIA-MS, FAIMS, feces, depression

## Abstract

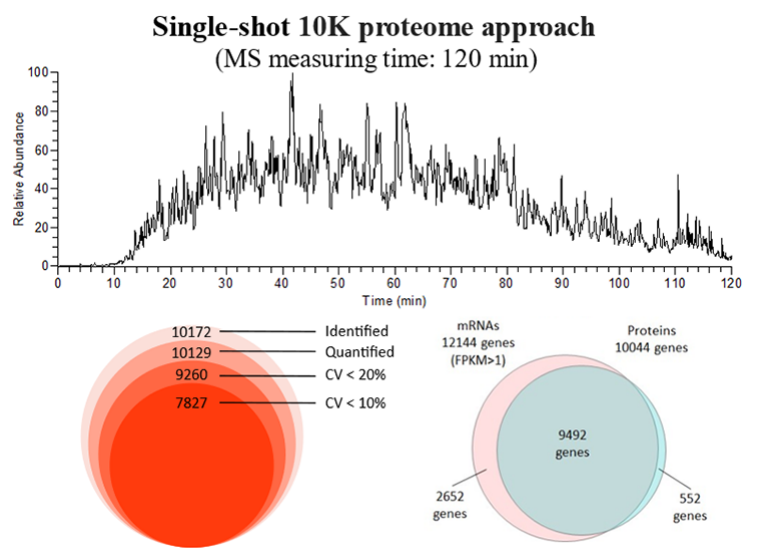

The evolution of mass spectrometry (MS) and analytical techniques has led to the demand for proteome analysis with high proteome coverage in single-shot measurements. Focus has been placed on data-independent acquisition (DIA)-MS and ion mobility spectrometry as techniques for deep proteome analysis. We aimed to expand the proteome coverage by single-shot measurements using optimizing high-field asymmetric waveform ion mobility spectrometry parameters in DIA-MS. With our established proteome analysis system, more than 10,000 protein groups were identified from HEK293 cell digests within 120 min of MS measurement time. Additionally, we applied our approach to the analysis of host proteins in mouse feces and detected as many as 892 host protein groups (771 upregulated/121 downregulated proteins) in a mouse model of repeated social defeat stress (R-SDS) used in studying depression. Interestingly, 285 proteins elevated by R-SDS were related to mental disorders. The fecal host protein profiling by deep proteome analysis may help us understand mental illness pathologies noninvasively. Thus, our approach will be helpful for an in-depth comparison of protein expression levels for biological and medical research because it enables the analysis of highly proteome coverage in a single-shot measurement.

## Introduction

Proteins are the end products of genes and are molecules with various functions. Although it is best to measure the protein accumulation level by measuring the protein itself, protein expression prediction by transcriptome analysis is frequently conducted because proteins are transcribed from messenger RNA (mRNA). This is often attributed to the fact that more molecules can be detected in transcriptome analysis than in proteome analysis. However, it has been reported that the correlation between mRNA and protein expression levels is not high, around a correlation coefficient *r* = 0.6,^1−3^ which inevitably increases the significance of measuring the protein amounts themselves in the multiomics era. Therefore, there is a need for further improvement in the simplicity and depth of proteome analysis.

An effective way to extend the depth of analysis in proteome analysis is to fractionate digested peptides with strong cation exchange (SCX) chromatography, high-pH C18, or hydrophobic interaction liquid chromatography (HILIC) and measure using liquid chromatography with tandem mass spectrometry (LC–MS/MS).^4−6^ For quantification, labeling with isobaric tagging methods, such as a tandem mass tag (TMT) or iTRAQ before peptide fractionation, helps evade the issue of fractionation reproducibility and allows multiplexed measurements using LC–MS/MS, enabling comparative quantification of 8000–10,000 proteins.^3,5,7^ However, isobaric tagging reagents are costly, and the number of specimens that can be labeled is limited (18 plex is the maximum for commercial products). To solve these problems, single-shot label-free proteomics by data-independent acquisition (DIA)-MS has recently come into the limelight. DIA-MS is highly quantitative, allows for deep analysis, and does not require expensive labeling reagents.^8−10^ In fact, we have successfully identified and quantified more than 8000 proteins from mammalian cells by a single-shot measurement using DIA-MS.^11^ The disadvantage of DIA-MS is that it requires the generation of a spectral library from premeasured MS data during protein identification analysis. Still, with the development of Prosit, a high-quality spectral library can be generated from a protein sequence database, eliminating the need for premeasurement for the generation of a spectral library.^12^ Considering these improvements, the convenience of label-free proteome analysis by DIA-MS is close to becoming almost equal to that of the conventional data-dependent acquisition (DDA)-MS.

In DIA-MS, quadrupole (Q)-time of flight-MS or Q-Orbitrap MS is generally used. In reports of over 7000 proteins identified by DIA-MS, most of them are Q-Orbitrap MS.^11,13,14^ In the case of Q-Orbitrap MS, the molecules that pass through Q are fragmented, and the ions are accumulated by the ion routing multipole and detected by Orbitrap MS. This accumulation by the ion routing multipole is a great advantage for measuring trace molecules, and the Q-Orbitrap MS enables measurement with a high analytical depth. More recently, it has become possible to attach high-field asymmetric waveform ion mobility spectrometry (FAIMS) to the front end of Q-Orbitrap MS, which reduces the chemical background noise and ion complexity in ion mobility separation.^15^ FAIMS is expected to further expand protein identifications.

In this study, the parameters of FAIMS were optimized for DIA-MS to improve the analytical depth of the simple single-shot proteome analysis. Additionally, our system was applied to the fecal proteome analysis to reveal alterations in fecal host proteins due to repeated social defeat stress (R-SDS), a mouse model used to study depression.

## Materials and Methods

### Animal Study

Nine week old male C57BL/6N and Institute of Cancer Research (ICR) mice (retired from breeding) were obtained from Japan SLC (Shizuoka, Japan). Mice were housed in groups of 4–5 mice per cage in a specific pathogen-free, temperature- and humidity-controlled vivarium under a 12 h light/12 h dark cycle (light, 6:00 a.m. to 6:00 p.m.) with food and water available *ad libitum* for at least 1 week before the experiments. The procedures for animal care and use were in accordance with the National Institutes of Health Guide for the Care and Use of Laboratory Animals and were approved by the Animal Care and Use Committees of the Kobe University Graduate School of Medicine.

The mice were subjected to R-SDS and behavioral tests, including the social interaction test and the elevated plus-maze test, as previously described,^16,17^ with minor modifications. Before R-SDS, ICR male mice were screened for their aggressiveness against a different C57BL/6N mouse for 3 min daily for 3 days. We evaluated aggression by measuring the latency of the first attack and the number of attacks during this period. We used the ICR mice that exhibited stable aggression for further experiments. A week before the R-SDS, the male C57BL/6N mice were singly housed and kept throughout the experiments. These mice were then transferred to the home cage of a male ICR mouse and were defeated for 10 min daily for 10 consecutive days. The pairs of defeated and aggressor mice were randomized daily to minimize the variability in the aggressiveness of the aggressor mice. After the 10 min defeat episode, the defeated mice were returned to their home cages until they were subjected to the next bout of R-SDS the next day. The control mice were also singly housed but left untreated.

The defeated and control mice were subjected to two behavioral tests, namely, the social interaction test and the elevated plus-maze test. In the social interaction test, all mice first received habituation to the behavioral chamber before R-SDS. After R-SDS, the defeated and control mice were placed in the chamber with a novel ICR mouse enclosed in a metal meshwork placed at one end of the chamber. The experimental mice were allowed to explore the chamber for 150 s freely. The area at the opposite side of the metal meshwork was defined as an avoidance zone, and the time spent in this zone was measured as an index for depression-like behaviors. As to the elevated plus-maze test, the defeated and control mice were placed on an end of either of the closed arms of the behavioral maze apparatus, which consisted of two open arms and two closed arms. The mice were allowed to freely explore the maze for 5 min, and the time spent in the open arms was an index for anxiety. Less time spent in the open arms is considered as heightened anxiety.

After behavioral tests, the defeated mice received another episode of stress the next day and were then subjected to sample collection. The defeated and control mice were anesthetized with an intraperitoneal injection of sodium pentobarbital (100 mg/kg, Nacalai Tesque, Kyoto, Japan) and transcardially perfused with a flush of saline. The large intestine was excised, and the feces inside was collected. The feces was frozen and maintained at −80 °C until use.

### Protein Extraction

Proteins in the HEK293 cell sample were extracted in 100 mM Tris-HCl (pH 8.5) containing 2% sodium dodecyl sulfate (SDS) by sonication using Bioruptor II (CosmoBio, Tokyo, Japan) for 10 min. Protein extraction from feces with a focus on the host proteins was performed with a slight modification in the previously reported procedure.^18^ Briefly, the feces was added in tris-buffered saline (TBS) with protease inhibitors [cOmplete ULTRA Tablets (CAT# 5892970001), Sigma-Aldrich, MO, USA], and soluble proteins were extracted by pipetting with a tip with the tip cut off and inverting after incubating for 30 min on ice. After centrifugation at 15,000*g* for 15 min at 4 °C to remove the pellet (bacteria and food debris), the supernatant was transferred to a fresh tube and subjected to trichloroacetic acid precipitation (12.5% v/v, final concentration of trichloroacetic acid), followed by two acetone washings. The sample was redissolved in 100 mM Tris-HCl (pH 8.5) containing 2% SDS by sonication using Bioruptor II (CosmoBio). Protein concentration in the protein extract was determined using a BCA protein assay kit (CAT# 23225, Thermo Fisher Scientific) and adjusted to 1 μg/μL with 100 mM Tris-HCl (pH 8.5) containing 2% SDS.

### Protein Digestion

20 μL of the protein extract was treated with 20 mM tris(2-carboxyethyl)phosphine at 80 °C for 10 min and alkylated using 35 mM iodoacetamide at room temperature for 30 min while being protected from light and subjected to clean up and digestion with single-pot solid phase-enhanced sample preparation (SP3)^19,20^ with minor modifications. Briefly, two types of Sera-Mag SpeedBead carboxylate-modified magnetic particles (hydrophilic particles, CAT# 45152105050250; hydrophobic particles, CAT# 65152105050250; Cytiva, Marlborough, MA, USA) were used. These beads were combined at a 1:1 (v/v) ratio, washed twice with distilled water, and reconstituted in distilled water at a concentration of 15 μg solids/μL. 20 μL of reconstituted beads was then added to the alkylated protein sample followed by 99.5% ethyl alcohol to bring the final concentration to 75% (v/v), with mixing for 5 min. The supernatant was discarded, and the pellet was washed with 80% ethyl alcohol and 100% acetonitrile (ACN). The beads were then resuspended in 100 μL of 50 mM Tris-HCl (pH 8.0) with 1 μg of trypsin/Lys-C Mix (CAT# V5072, Promega, Madison, WI, USA) and mixed gently at 37 °C overnight to digest proteins. The digested sample was acidified with 20 μL of 5% trifluoroacetic acid (TFA) and then sonicated with Bioruptor II (CosmoBio) at a high level for 5 min at room temperature. The sample was desalted using a STAGE tip (CAT# 7820-11200, GL Sciences Inc., Tokyo, Japan) according to the manufacturer’s protocol, followed by drying in a centrifugal evaporator (miVac Duo concentrator, Genevac Ltd., Ipswich, UK) and redissolving in 2% ACN containing 0.1% TFA. The peptide concentration in the redissolved sample was determined using a Lunatic instrument (Unchained Labs, Pleasanton, CA, USA) and transferred to a hydrophilic MS vial [ProteoSave vial (CAT# 11-19-1021-10); AMR Inc., Tokyo, Japan].

### DIA-MS-Based Proteomics

The peptides were directly injected onto a 75 μm × 20 cm PicoFrit emitter (CAT# PF360-75-8-N-5, New Objective, Woburn, MA, USA) packed in-house with C18 core–shell particles [CAT# 51227 (disassembled the column and got the particles), CAPCELL CORE MP 2.7 μm, 160 Å material; Osaka Soda, Osaka, Japan] at 50 °C and then separated using a 60 min gradient at 100 nL/min using an UltiMate 3000 RSLCnano LC system (Thermo Fisher Scientific). Details of the nanoLC program are given in Table S1. Peptides eluted from the column were analyzed using an Orbitrap Exploris 480 mass spectrometer (Thermo Fisher Scientific) for overlapping window DIA-MS.^11,21^ The MS1 spectra were collected in the range of *m*/*z* 395–645, 495–745, 395–765, or 495–865 at 30,000 resolution to set an automatic grain control (AGC) target of 3 × 10^6^. MS2 spectra were collected at 200–1800 *m*/*z* at 30,000 resolution to set an AGC of 3 × 10^6^, a maximum injection time of “auto”, and stepped normalized collision energies of 22, 26, and 30%. The overlapping window patterns at *m*/*z* 400–640 or *m*/*z* 500–740 (isolation window width, 4 Da) and *m*/*z* 400–760 or *m*/*z* 500–860 (isolation window width, 6 Da) were used for window placements optimized *via* Skyline v4.1 (Table S2).^22^ In FAIMS, five conditions of coefficient of variation (CV) values (−40, −45, −50, −55, −60 V) and five conditions of inner temperature (IT) and outer temperature (OT) settings in FAIMS (IT 100 °C/OT 80 °C, IT 100 °C/OT 90 °C, IT 100 °C/OT 100 °C, IT 90 °C/OT 100 °C, IT 80 °C/OT 100 °C) were tested.

In deep proteome analysis, the peptides were directly injected into a 75 μm × 40 cm PicoFrit emitter (New Objective) packed in-house with C18 core–shell particles (CAPCELL CORE MP 2.7 μm, 160 Å material; Osaka Soda) at 50 °C and then separated with a 120 min gradient at 100 nL/min. Details of the nanoLC program are provided in Table S1. In overlapping window DIA-MS parameters, MS1 spectra were collected in the range of *m*/*z* 495–745 at 15,000 resolution to set an AGC target of 3 × 10^6^. MS2 spectra were collected at *m*/*z* 200–1800 at 45,000 resolution to set an AGC target of 3 × 10^6^, a maximum injection time of “auto”, and stepped normalized collision energies of 22, 26, and 30%. The width of the isolation window was set to 4 Da, and overlapping window patterns at *m*/*z* 500–740 were used for window placements optimized *via* Skyline 4.1 (Table S2). The CV value and OT/IT on FAIMS were −45 V and IT 90 °C/OT 100 °C, respectively.

The LC–MS/MS files (.raw) were searched against human or mouse spectral libraries using Scaffold DIA (Proteome Software, Inc., Portland, OR, USA). These spectral libraries were generated from the human protein sequence database (proteome ID UP000005640, reviewed, canonical, 20,381 entries) and the mouse protein sequence database (proteome ID UP000000589, reviewed and unreviewed, canonical, 55,366 entries) by Prosit.^12,23^ The search parameters on the Scaffold DIA were as follows: experimental data search enzyme, trypsin; maximum missed cleavage sites, 1; precursor mass tolerance, 10 ppm; fragment mass tolerance, 10 ppm; static modification; and cysteine carbamidomethylation. The threshold for protein identification was set such that both protein and peptide false discovery rates (FDRs) were <1%. The protein and peptide quantification values were calculated using the Scaffold DIA. The protein quantification data were transformed to log_2_ (protein intensities) and filtered so that for each protein, at least one group contained a minimum of 70% valid values. The remaining missing values were imputed by random numbers drawn from a normal distribution (width, 0.3; downshift, 2.8) in Perseus v1.6.15.0.^24^ The thresholds for changed protein groups were a 2-fold change or more and *p* < 0.05 (Welch *t*-test) that differed between the two groups. Enrichment analysis of disease ontology (based on the HumanPSD database) was conducted using the geneXplain platform (geneXplain GmbH, Wolfenbüttel, Germany). Gene ontology (GO) enrichment analysis was carried out by DAVID (https://david.ncifcrf.gov/).^25^

To validate the protein identification results using search engines other than Scaffold DIA, the LC–MS/MS file (.raw) was transformed to a mzML file by ProteoWizard’s MSconvert (version: 3.0.19254).^26^ The MSconvert parameters were as follows: peakPicking, vendor msLevel = 1-; demultiplex, optimization = overlap_only; and massError = 10.0 ppm. The mzML file was converted into a .dia file using DIA-NN (version: 1.8, https://github.com/vdemichev/DiaNN).^27^ The peptide and protein were identified from the dia file by a two-step search using DIA-NN. First, the spectral library for the library-free search was generated from the human protein sequence database (proteome ID UP000005640, reviewed, canonical, 20,381 entries) using DIA-NN. The DIA-NN search parameters were as follows: peptide length range, 7–45; precursor charge range, 2–4; precursor *m*/*z* range, 490–750; fragment ion *m*/*z* range, 200–1800; mass accuracy, 10 ppm; MS1 accuracy, 10 ppm; static modification, cysteine carbamidomethylation; and “remove likely interferences”, “use isotopologues”, and “unrelated runs” were enabled. Additional commands entered into the DIA-NN command line were as follows: --peak-translation and --relaxed-prof-inf. The protein identification threshold was set at 1% or less for both peptide and protein FDRs. The second search analyzed the abovementioned setting again using a specific spectral library generated from the first search.

## Results and Discussion

### Assessment of FAIMS Parameters for DIA-MS

**Figure 1:**
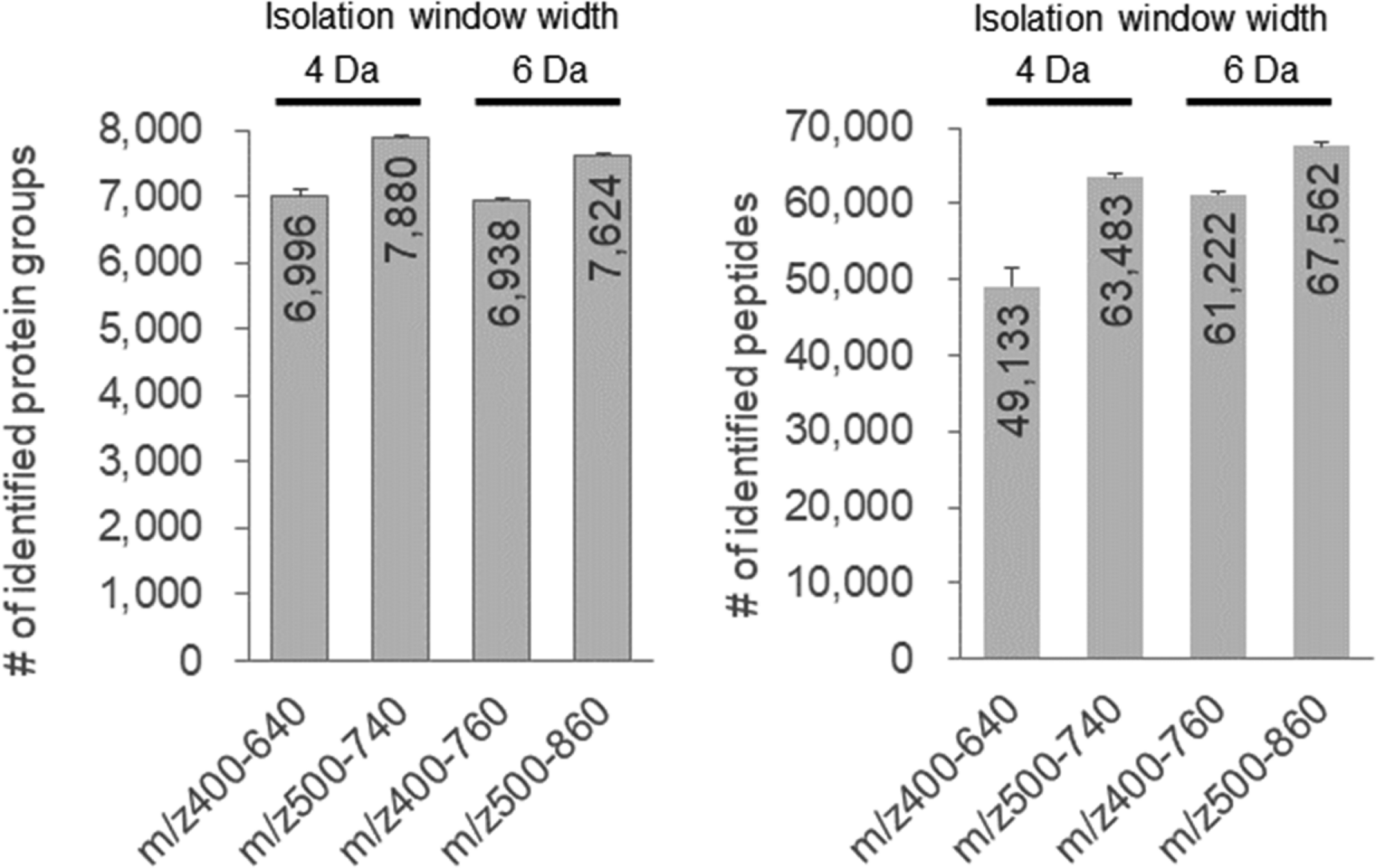
Number of protein groups and peptides identified using the four DIA-MS parameters for 200 ng of the HEK293 cell tryptic digest. DIA-MS in the ranges of *m*/*z* 400–640 or *m*/*z* 500–740 and *m*/*z* 400–760 or *m*/*z* 500–860 was set to a window width of 4 and 6 Da, respectively.

First, we examined DIA-MS parameters to obtain high proteome coverage by DIA-MS (Figure 1). Four parameters with the same DIA-MS cycle time were compared. The highest number of peptides identified was in the DIA-MS parameter of *m*/*z* 500–860, and the highest number of protein groups identified was in the DIA-MS parameter of *m*/*z* 500–740. Compared to the *m*/*z* 500–740 parameter, the *m*/*z* 500–860 parameter detected peptides in a wider range of *m*/*z*, thus increasing the number of identified peptides. Meanwhile, in the *m*/*z* 500–740 parameter, the narrow isolation window of 4 Da reduced the type of precursor ions that can pass through the Q at one time; however, the amount of fragment ions accumulated per ion type passed through can be increased for the same parameters of the AGC target and maximum injection time. This has allowed us to identify more trace peptides and increase the number of identified proteins. Since this study aims to expand proteome coverage, the DIA-MS parameter of *m*/*z* 500–740 was adopted.

**Figure 2:**
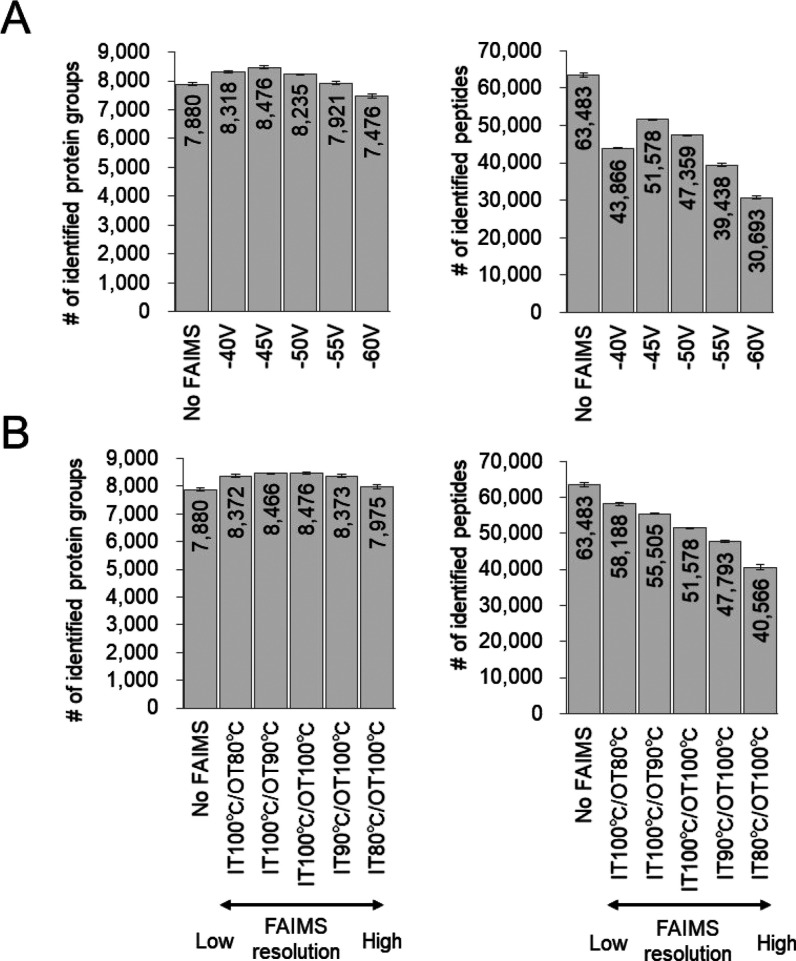
Comparison of FAIMS parameters in DIA-MS. (A) Number of protein groups and peptides identified using FAIMS at the compensation voltages from −40 to −60 V in 200 ng of the HEK293 cell tryptic digest. (B) Number of protein groups and peptides identified by FAIMS at the different ion mobility resolution in 200 ng of the HEK293 cell tryptic digest. The ion mobility resolution of FAIMS varies with the temperature change between IT and OT; IT 100 °C/OT 100 °C is the manufacturer’s default standard resolution.

In FAIMS in DDA-MS, it is common to make measurements by switching between two and three compensation voltage values,^28^ but in DIA-MS, a cycle time of MS is longer, so it is necessary to limit the compensation voltage setting to 1 for high-sensitivity analysis. We investigated the optimal compensation voltage value at which the protein identification constant is extended in DIA-MS (Figure 2A). From compensation voltages of −40 to −55 V, more proteins were identified than those without FAIMS, and −45 V had the highest number of protein identifications. At the peptide level, no FAIMS had the highest number of peptides identified, but from −40 to −60 V, −45 V had the highest number of peptides identified. Based on these results, the optimal value of the compensation voltage of FAIMS in DIA-MS was set to −45 V. FAIMS reduced the complexity of the peptide mixture, resulting in a decrease in the number of peptides identified. Still, as a trade-off, trace peptides were detected, resulting in an increase in the number of proteins identified. The optimal value of the compensation voltage of −45 V for DIA-MS was consistent with that reported by Bekker-Jensen *et al.*(15) and is considered reasonable.

Next, we investigated the optimal resolution of FAIMS in DIA-MS (Figure 2B), and the resolution of FAIMS was set by varying the IT and OT in FAIMS. The number of identified proteins was higher in IT 100 °C/OT 90 °C and IT 100 °C/OT 100 °C at the same level. The number of peptides identified increased as the resolution decreased. Since IT 100 °C/OT 90 °C was clearly higher in terms of the number of peptides identified compared to IT 100 °C/OT 100 °C, IT 100 °C/OT 90 °C was chosen as the optimal setting for FAIMS resolution. As proteome analysis by DIA-MS can identify peptides from chimeric spectra, it can be inferred that the rather wide ion uptake by FAIMS did not affect the identification of proteins and increased the number of identified peptides. Meanwhile, the entire FAIMS transmission profile may shift when the electrode temperatures are changed; however, the effect was small because >90% of the peptides identified at the standard IT 100 °C/OT 100 °C were included in the peptides identified at IT 100 °C/OT 90 °C (Figure S1).

From these results, the best FAIMS parameters for DIA-MS were a compensation voltage value of −45 V and a FAIMS resolution setting of IT 100 °C/OT 90 °C.

### Ultradeep Proteome Analysis by DIA-MS with FAIMS

**Figure 3:**
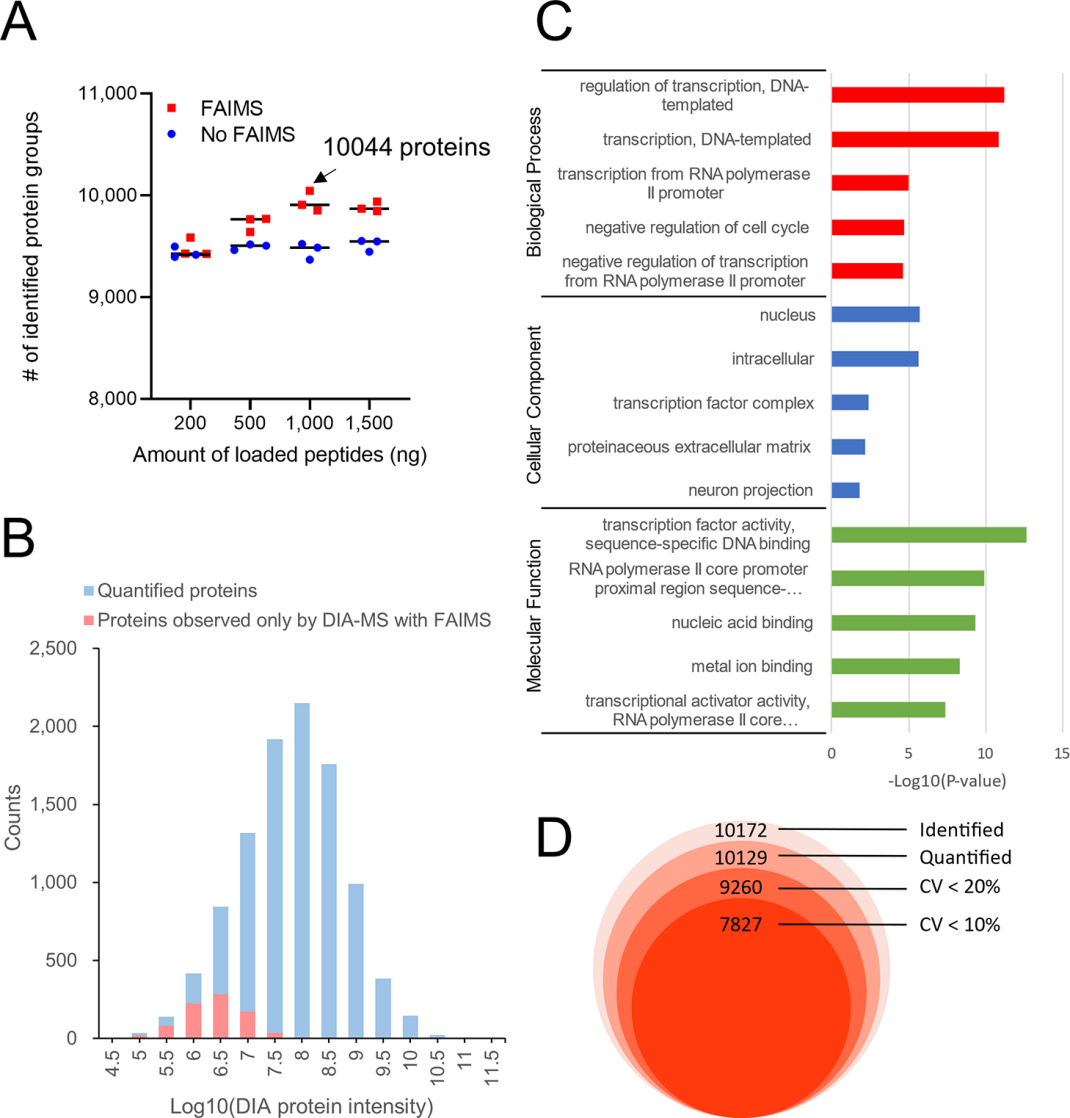
Ultradeep proteome analysis by DIA-MS with FAIMS in a single-shot measurement. (A) Comparison of the loading volume with and without FAIMS in the HEK293 cell tryptic digest. (B) Histogram of the log_10_ DIA protein intensity observed in proteome analysis by DIA-MS with FAIMS in 1000 ng of the HEK293 cell tryptic digest. Quantified proteins are indicated by blue bars. Proteins that were not observed by DIA-MS without FAIMS in 1000 ng of the HEK293 cell tryptic digests are indicated by red bars. (C) GO enrichment analysis of proteins observed with only DIA-MS with FAIMS [proteins indicated by red bars in (B)]. (D) The number of protein groups identified by triplicate DIA-MS with FAIMS and the number of protein group CVs below the defined thresholds were calculated in 1000 ng HEK293 cell tryptic digest.

In the study of FAIMS parameters, DIA-MS analysis was conducted in 60 min using a 20 cm column; here, DIA-MS analysis was conducted in 120 min using a 40 cm long column (for a deep proteome analysis system) to expand proteome coverage. The parameters of FAIMS used were optimized for DIA-MS. Figure 3A shows the change in loading volume with and without FAIMS. When the loading volume was 200 ng of the HEK293 cell digest, there was no change in the number of proteins identified with and without FAIMS, but increasing the loading volume increased the number of proteins identified with FAIMS. The deep proteome analysis system showed no change in the number of proteins identified with 200 ng of the digest as LC enhanced the separative power and reduced the benefit of FAIMS in reducing the complexity of the peptides. However, increasing the loading volume increased the complexity of the peptides to be ionized, which benefited from FAIMS. As for the appropriate loading volume for the deep proteome analysis using FAIMS, a loading volume of 1000 ng was sufficient. This is because there was no increase in the number of proteins identified at 1500 ng compared to those at 1000 ng. For analysis of longer gradients, a loading volume of 1000 ng or more may be appropriate. We were able to identify up to 10,044 protein groups (protein FDR < 1%, peptide FDR < 1%) using Scaffold DIA software when 1000 ng of the digest was analyzed using DIA-MS with FAIMS (Table S3). The same MS data were also analyzed using DIA-NN, and 10,716 protein groups (protein FDR < 1%, peptide FDR < 1%) were identified (Figure S2). The 9865 protein groups identified by each search engine overlapped, and there were few differences among the search engines. The single-shot 10K proteome approach (here, we are calling the approach that observed more than 10,000 proteins the“10K proteome approach”) has been reported by Muntel *et al.* in 6 h of the DIA-MS measurement from human testis tissue^13^ and Meier *et al.* in 100 min of the BoxCar acquisition method from the mouse cerebellum.^29^ The BoxCar acquisition method needs to be measured beforehand for spectral library generation, and they conducted DDA-MS measurements of 24 fractions by high-pH fractionation for 100 min each. We generated a spectral library from the human protein sequence database (20,381 entries) using Prosit. We were the first to identify more than 10,000 proteins from a single cell type in an MS measurement time of 120 min or less without prior MS measurements for spectral library generation. In the analysis of 1000 ng of the HEK293 cell tryptic digest, most of the proteins detected by DIA-MS with FAIMS instead of DIA-MS alone were in the low protein intensity range (Figure 3B), and GO enrichment analysis of these proteins showed that many of them were transcription factor-related terms (Figure 3C), indicating that DIA-MS with FAIMS is useful for observing trace amounts of transcription factor-related proteins. Figure 3D shows the reproducibility of the protein intensities in our system. The total number of identified proteins was 10,172, and more than 9000 proteins were included even at CV < 20%. From these results, we succeeded in establishing a reproducible single-shot 10K proteome approach.

**Figure 4:**
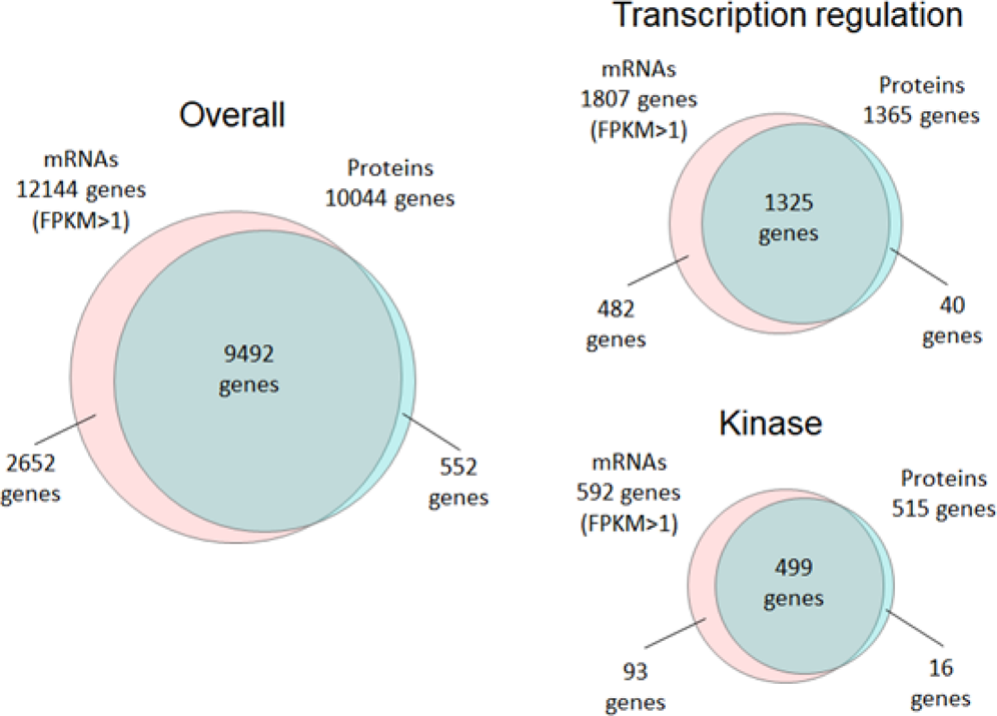
Venn diagram showing the overlap of the genes observed by proteome analysis (protein level) and RNA-seq (mRNA level) in HEK293 cells. For the proteome analysis of HEK293 cells, the data in which 10,044 protein groups were identified in this study were utilized, and for the RNA-seq of HEK293 cells, previously reported data were utilized.^2^ Categories of “transcription regulation” and “kinase” were extracted from Uniprot Keyword.

We then compared the number of genes detected by RNA-seq [fragments per kilobase million (FPKM) > 1] using the proteome data where most proteins were identified (Figure 4). Although the number of genes observed *via* RNA-seq was larger than that observed *via* proteome analysis, 78% of the genes observed *via* RNA-seq could be observed *via* single-shot proteome analysis. The similar trend was observed for transcription regulation proteins and kinases, which are known to be trace proteins. When Nagaraj *et al.* compared large-scale proteome analysis with RNA-seq in 2011, they identified 10,255 proteins in a total MS measuring time of 12 days (288 h).^3^ Our 10K proteome approach reduced the time required to obtain comparable protein profiling results from 12 days to 2 h, showing that the proteome analysis technology has evolved significantly in about 10 years.

### Proteomic Analysis of Fecal Host Proteins in a Mouse Model of R-SDS

Although feces has been used to analyze the intestinal microbiota and their metabolites, host proteins in the feces have not been sufficiently analyzed due to the abundance of food- and bacteria-derived proteins in addition to host proteins. Since the gastrointestinal tract expels the feces, the feces may include host proteins derived from gastrointestinal tissues. These proteins may be useful for new biomarkers for various diseases with gastrointestinal dysfunctions.^18,30^ Stress due to adverse and demanding conditions alters the functions of the brain and peripheral organs, such as the gastrointestinal organs, and is thought to precipitate various diseases including depression.^31,32^ Rodent studies with chronic stress models, including the R-SDS, have revealed neural and non-neural mechanisms of stress-induced depression-related behaviors.^17,33−37^ In some studies, chronic stress altered gut microbiota profiles, contributing to depression-related behaviors.^38−40^ Clinical studies have shown altered microbiota profiles in the feces of depressive patients.^41,42^ However, how gut microbiota affects neural functions remains poorly understood, and the detection of fecal host proteins may provide a hint for elucidating the interaction between altered microbiota and host tissues. Thus, we considered that a deep proteome analysis would be suitable for observing changes in host-derived proteins in fecal samples containing abundant bacteria- and food-derived proteins and examined changes in fecal host proteins of mice that received R-SDS using our 10K proteome approach.

In R-SDS, the mice received social defeat stress for 10 min daily for 10 consecutive days, and the depressive-like and anxiety-like behaviors of these stressed mice and control mice were evaluated using two behavioral tests, namely, the social interaction test and the elevated plus-maze test (Figure S3A,B). In the social interaction test, the stressed mice spent more time in the avoidance zone relative to the control mice (Figure S3C: stressed mice = 54.9 ± 19.5%, control mice = 12.0 ± 4.37%), with a large interindividual variability, which is consistent with the previous literature.^17^ It has been known that some mice are resilient to the development of social avoidance, whereas others are susceptible. Therefore, we categorized the stressed mice that spent more than 50% of the total time in the avoidance zone as the avoidant and the others as the nonavoidant as previously described^17^ and found that R-SDS significantly increased the fraction of the avoidant mice (avoidant and nonavoidant mice were zero and five in the control group and were four and three in the R-SDS group; *P* = 0.0384 in χ^2^ test). In the elevated plus-maze test, R-SDS slightly decreased the time spent in the open arms but without statistical significance due to high interindividual variability (stressed mice = 25.9 ± 9.5%, control mice = 35.4 ± 13.0%).

**Figure 5:**
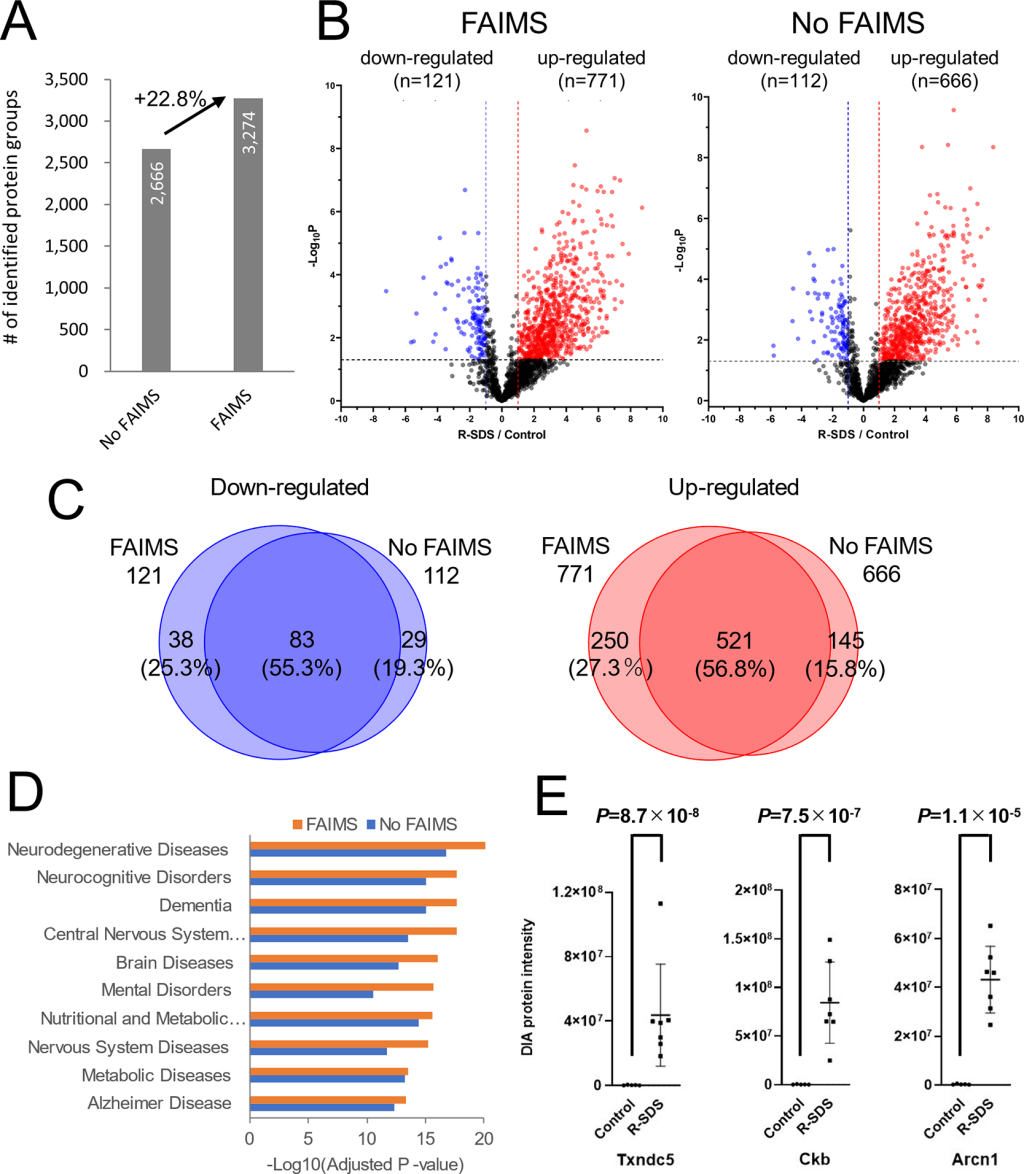
Changes in fecal host proteins in a mouse model of R-SDS determined by the ultradeep proteome analysis. (A) Number of protein groups identified using DIA-MS with and without FAIMS. (B) Volcano plot of the intensities of the host protein obtained from the feces of the R-SDS model and control mice. The red dots were proteins that were upregulated in the feces of the R-SDS model mice, and the blue dots were proteins that were downregulated in the feces of the R-SDS model mice. (C) Overlap between altered proteins in R-SDS feces detected by DIA-MS with FAIMS and those detected by DIA-MS without FAIMS. (D) Disease ontology enrichment analysis of host proteins upregulated in the feces of the R-SDS model mice. (E) Protein intensities of three representative proteins upregulated in the feces of the R-SDS model mice.

Figure 5A compares the number of protein groups identified using DIA-MS with and without optimized FAIMS. More proteins (+22.8%) were identified using DIA-MS with FAIMS than without FAIMS. The advantage of FAIMS was considerable in complex samples, such as feces, considering that in the case of 1000 ng of the HEK293 cell tryptic digest, the number of proteins identified was only approximately 5% higher when DIA-MS with FAIMS was performed than DIA-MS without FAIMS. With regard to changes in the intensities of the host protein obtained from the feces of stressed and control mice, DIA-MS with FAIMS detected 771 increasing and 121 decreasing proteins in the feces of stressed mice, and DIA-MS without FAIMS detected 666 increasing and 112 decreasing proteins in the feces of stressed mice (Figure 5B and Table S4). Therefore, more altered proteins were detected using DIA-MS with FAIMS. However, some protein changes were observed only in DIA-MS without FAIMS (Figure 5C). It was confirmed that some altered proteins were missed during ion selection by FAIMS. We then conducted disease ontology enrichment analysis on the proteins increased by R-SDS (Figure 5D). Since we could not find an extensive disease ontology database for the mice, we used the human disease ontology database instead. Interestingly, categories related to brain diseases that might be associated with R-SDS were extracted. These differences were more pronounced in DIA-MS with FAIMS, especially in ”mental disorders”, which is highly related to R-SDS, with a *P*-value of 2.1 × 10^16^ for DIA-MS with FAIMS and 2.1 × 10^11^ for DIA-MS without FAIMS. Although some proteins were lost due to FAIMS, deeper proteome analysis has led to the discovery of proteins that are highly relevant to the disease. Some of these proteins were annotated neuronally, suggesting that they were upregulated in the enteric nervous system by R-SDS and leaked into the feces. A principal component analysis segregated the proteomic patterns of the mice with and without R-SDS (Figure S4). Among the stressed mice, the avoidant and nonavoidant mice could not be clearly segregated using their fecal proteomic patterns, indicating that R-SDS altered the fecal host protein profiling irrespective of social avoidance development. These results suggest that fecal proteomic profiling is more sensitive to R-SDS than to social interactions. Although validation experiments with human specimens will be necessary in the future, the measurement of fecal host proteins is exploitable to identify noninvasive biomarkers for depression and other brain diseases associated with gut dysfunctions. Among the proteins altered in expression with R-SDS, thioredoxin domain-containing protein 5 (Txndc5), creatine kinase B-type (Ckb), and coatomer subunit delta (Arcn1), shown in Figure 5E, are included in the “mental disorders” category of disease ontology and changed in expression with R-SDS by more than 100-fold with a *P*-value of less than 0.00005. Thus, these proteins may be candidates for the fecal biomarkers of depression.

## Conclusions

To extend the proteome coverage in single-shot proteome analysis, FAIMS parameters in DIA-MS were optimized. Additionally, we were able to identify more than 10,000 protein groups by deep proteome analysis using DIA-MS with FAIMS, which is closer to the number of genes detected by RNA-seq. Applying our 10K proteome approach to host protein analysis in mouse feces, we could observe 892 variable proteins by R-SDS, a mouse model to study depression. Thus, the profiling of fecal host proteins by our 10K proteome approach may help us understand the pathology of various diseases associated with gastrointestinal alterations noninvasively.

## Supporting Information Available

Venn diagram of the number of peptides observed in the IT100T90 and IT100T100 parameters; comparison of the number of proteins identified using different search engines; mouse model of R-SDS; and principal component analysis of the fecal proteome data of the R-SDS model mice and control mice (PDF)
nanoLC program (XLSX)
DIA method for overlapping windows (XLSX)
List of proteins identified by overlapping window DIA with FAIMS in 1000 ng of in HEK293 cell tryptic digest (XLSX)
List of fecal host proteins that differ between R-SDS model and control mice (XLSX)

## Notes

Data Availability: The mass spectrometry proteomics data have been deposited to the ProteomeXchange Consortium *via* the jPOST partner repository^43^ with the dataset identifier PXD029853 and PXD032698 for ProteomeXchange and JPST001391 and JPST001537 for jPOST.
