# Supplementary material for: Single-Shot 10K Proteome Approach: Over 10,000 Protein Identifications by Data-Independent Acquisition-Based Single-Shot Proteomics with Ion Mobility Spectrometry
Source: J Proteome Res. 2022 May 6;21(6):1418–27. doi: 10.1021/acs.jproteome.2c00023 (PMC9171847; doi:10.1021/acs.jproteome.2c00023)
Supplement: Supplementary file 1 — pr2c00023_si_001.pdf [file pr2c00023_si_001.pdf]

Supporting information

Single-shot 10K proteome approach: Over 10,000 protein identifications by data independent acquisition based single-shot proteomics with ion mobility spectrometer

Yusuke Kawashima[1,*], Hirotaka Nagai[2], Ryo Konno[1], Masaki Ishikawa[1], Daisuke Nakajima[1], Hironori Sato[1], Ren Nakamura[1], Tomoyuki Furuyashiki[2], Osamu Ohara[1]

[1]Department of Applied Genomics, Kazusa DNA Research Institute, Kisarazu, Chiba 292-0818, Japan.
[2]Division of Pharmacology, Graduate School of Medicine, Kobe University, Chuo-ku, Kobe, 650-0017, Japan.
*Correspondence: Email: ykawashi@kazusa.or.jp; Tel: +81-438-52-3580, Fax.: +81-438-52-3501

Contents

Figure S1: Venn diagram of the number of peptides observed in the IT100T90 and IT100T100 parameters

Figure S2: Comparison of the number of identified proteins by different search engines

Figure S3: A mouse model of R-SDS

Figure S4: Principal component analysis of the fecal proteome data in R-SDS model mice and control mice.

Table S1: The nanoLC program (XLSX)

Table S2: DIA method for overlapping windows (XLSX)

Table S3: List of proteins identified by overlapping window DIA with FAIMS in 1000 ng of in HEK293 cell tryptic digest (XLSX)

Table S4: List of fecal host proteins that differ between R-SDS model and control mice (XLSX)

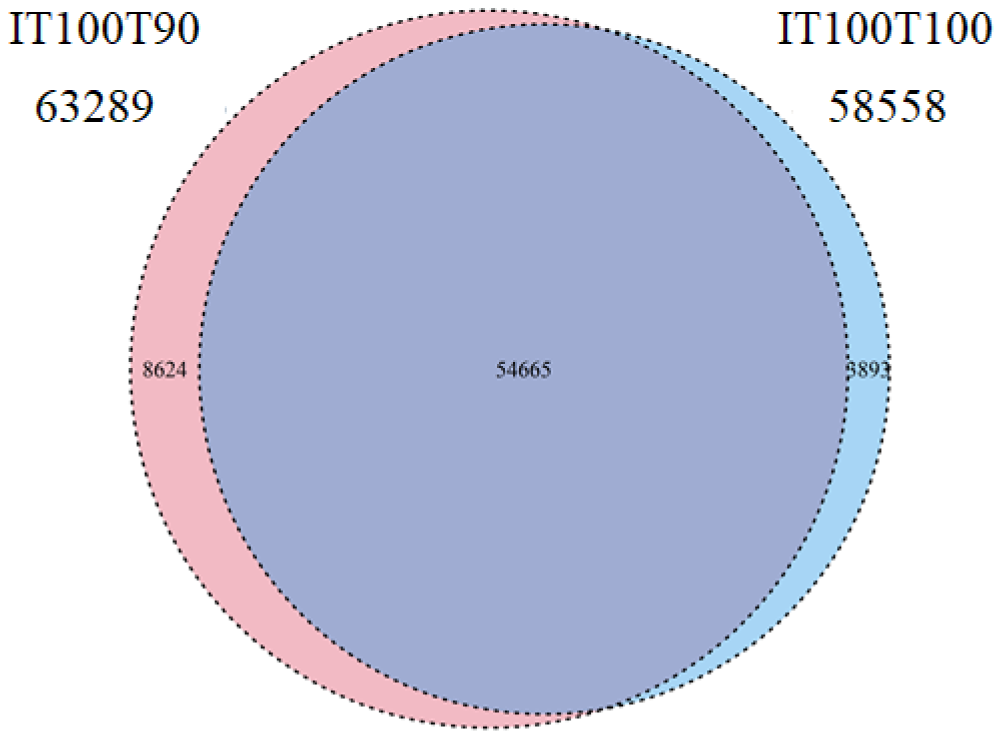

Figure S1. Venn diagram of the number of peptides observed in the IT100T90 and IT100T100 parameters.

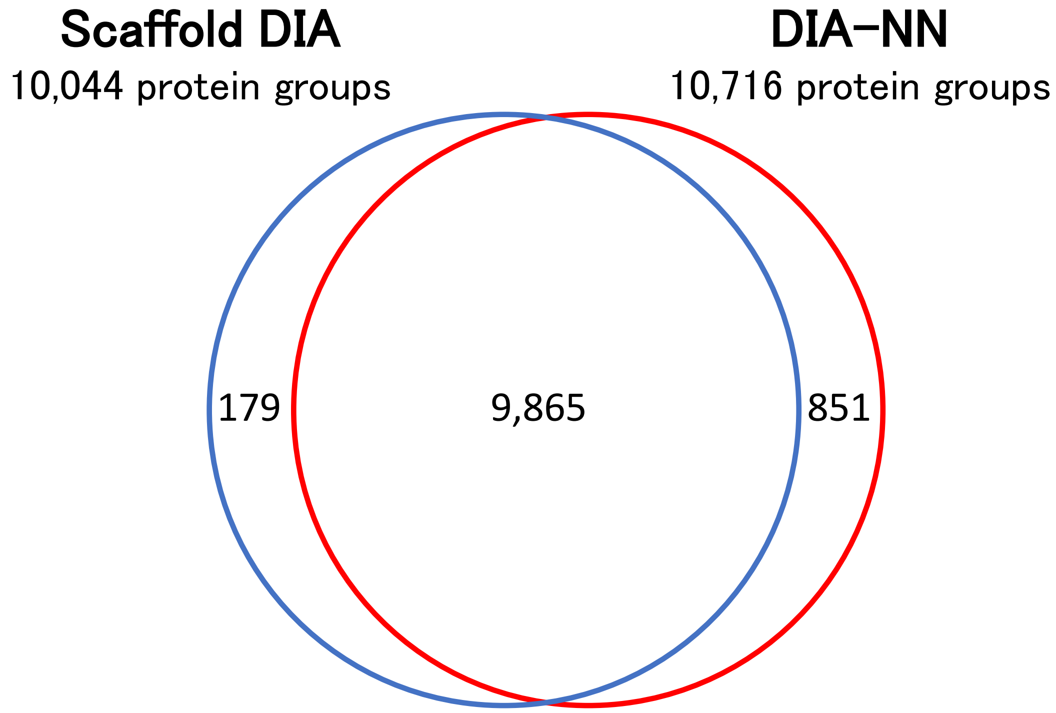

Figure S2. Comparison of the number of identified proteins by different search engines. The search engines were used Scafold DIA and DIA-NN.

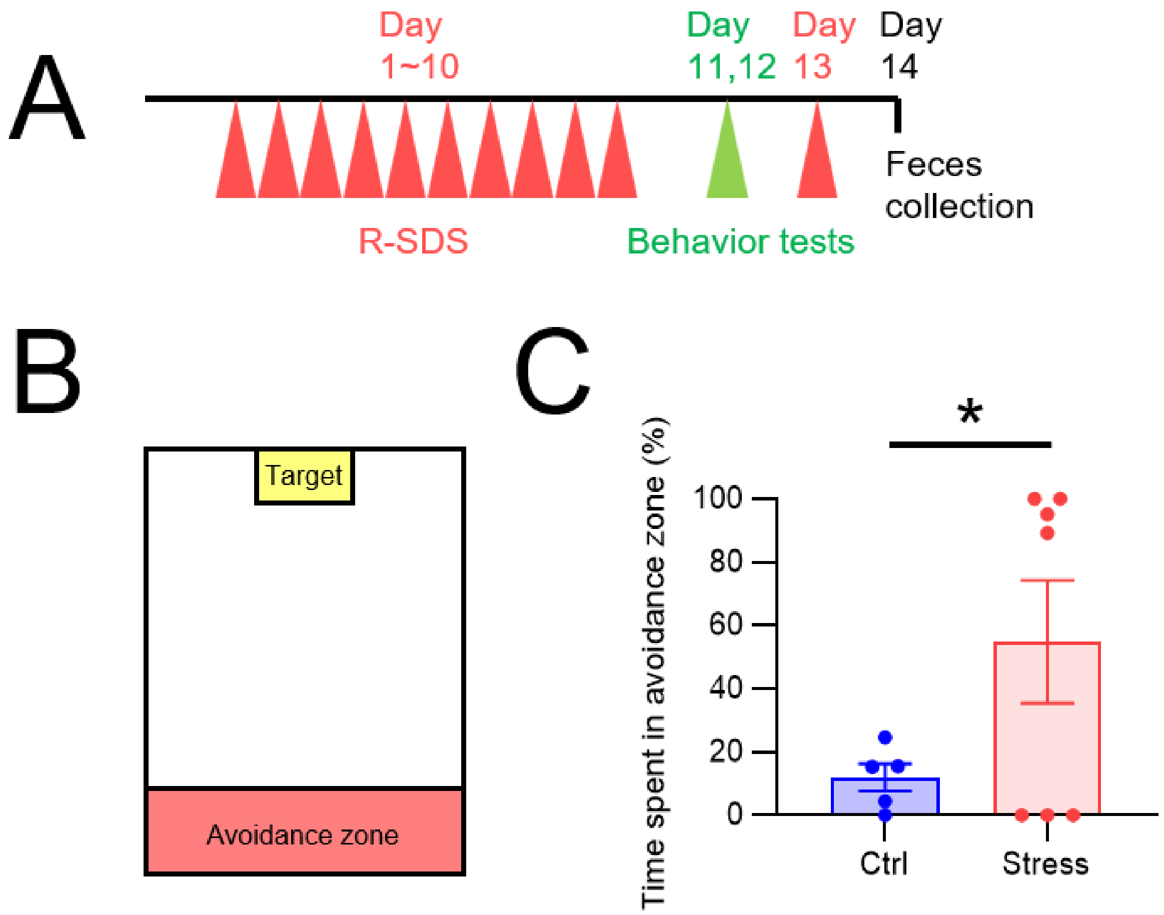

Figure S3. A mouse model of repeated social defeat stress (R-SDS). (A) The time schedule of animal experiments in this study. Male C57BL/6N mice received social defeat stress through an encounter with an aggressor Institute of Cancer Research (ICR) mouse for 10 min daily for 10 consecutive days, and the depressive-like and anxiety-like behaviors of these stressed mice and control mice were evaluated using two behavioral tests, namely the social interaction test and the elevated plus maze test. (B) A behavioral chamber used in social interaction test. After R-SDS, defeated and control mice were placed in the chamber with a novel ICR mouse enclosed in a metal meshwork placed at one end of the chamber. The experimental mice were allowed to freely explore the chamber for 150 s. The area at the opposite side of the metal meshwork was defined as an avoidance zone, and the time spent in this zone was measured as an index for depressive-like behaviors. (C) Time spent in the avoidance zone in the social interaction test. Values are expressed as means ± SEM. *$P < 0.05$ for Chi-square test (see Main text).

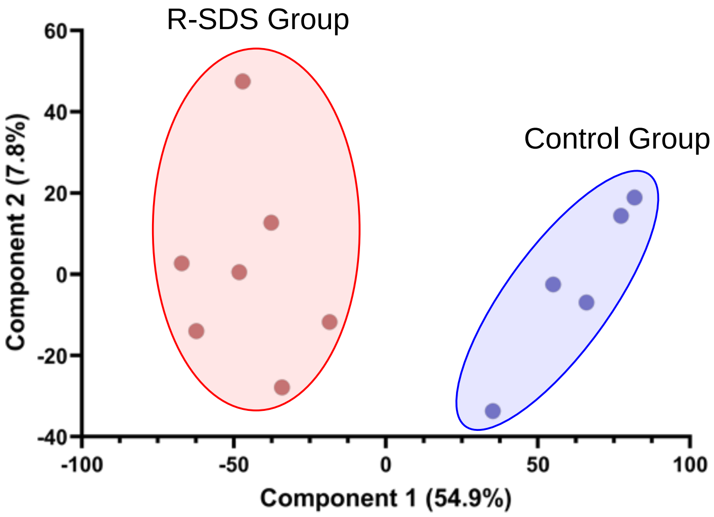

Figure S4. Principal component analysis of the fecal proteome data in R-SDS model mice and control mice.
